# Building infrastructures of abortion care in an un-caring state: acompañante’s carework and abortion access in Peru

**DOI:** 10.1086/723296

**Published:** 2023

**Authors:** Deirdre Duffy, Cordelia Freeman, Sandra Rodríguez

**Affiliations:** Faculty of Health Psychology and Social Care, Manchester Metropolitan University, Manchester; Department of Geography, University of Exeter. Amory Building, Rennes Drive, Exeter, EX4 4RJ; Pontificia Universidad Católica del Perú

## Abstract

For abortion seekers, Peru is an uncaring state where legal and policy interventions have resulted in violence, persecution, and neglect. This state of abortion uncare is set within historic and ongoing denials of reproductive autonomy, coercive reproductive care, and the marginalisation of abortion. Abortion is not supported, even where legally permissible. Here we explore abortion care activism within the Peruvian context, foregrounding a key mobilisation that has emerged against a state of un-care - *acompañante* carework. Through interviews with people involved in abortion access and activism in Peru, we argue that *acompañantes* have constructed an infrastructure of abortion care in Peru through the bringing together of actors, technologies, and strategies. This infrastructure is shaped by a feminist ethic of care that differs from minority world care assumptions regarding high quality abortion care in three key ways: (i) care is provided beyond the state; (ii) care is holistic; and (iii) care is collective. We argue that US feminist debates relating to the emerging hyperrestrictive state of abortion un-care as well as broader research on feminist care can learn from *acompañante* activism strategically and conceptually.

## 1 Introduction

It is May 2022 and we are currently witnessing the entrenchment of a hyperrestrictive legal environment for abortion in the United States(Murray 2021). Reproductive justice advocates have contended that the latest interventions are only part of the picture and the state of abortion uncare has been shaped through a range of micro- and macro-political and legal interventions. Black, indigenous and people of colour (BIPOC) and socio-economically disadvantaged communities have witnessed the steady encroachment of their pathways to abortion care through disinvestment in public health services and the targeted regulation of abortion providers or TRAP laws(Austin and Harper 2018; Solazzo 2019). That said, the *Dobbs vs. Jackson Women’s Health Organisation* judgement, if Justice Samuel Alito’s leaked brief is accurate, is a substantial escalation. It signals the predominantly conservative Supreme Court’s intention, through *Dobbs,* to overturn *Roe vs. Wade,* the 1973 judgement positioned as the fundamental federal protection of abortion care (Hannan, 2021). In *Roe’s* absence, legislatures in eleven states will be able to implement bills recently approved at a state-level which criminalise abortion provision and access to care without breaching federal protections(Silberner 2022).

In the immediate aftermath of the Alito leak, feminist pro-choice and reproductive justice activists have begun to openly consider how movements in jurisdictions where abortion has been heavily restricted have disrupted barriers to abortion. Within these discussions, Latin America is frequently used as a point of reference. Latin America has historically and continues to be home to some of the most restrictive abortion laws, policies, and regulations in the world (Fernández Anderson 2020). In most countries, abortion is only permitted under very specific circumstances: when necessary to save the mother’s life or health and in cases of rape. Some countries ban it without exception and criminalize those who have ‘unexplained’ miscarriages (Bergallo, Jaramillo Sierra and Vaggione 2018). Millions of clandestine abortions take place every year in Latin America in contexts where abortion is illegal or heavily restricted with prosecution and incarceration a very real threat (Dzuba, Winikoff and Peña 2013). At the same time, the period since the early 2000s has seen significant liberalisation of abortion law in Latin America. The achievements of activists in Colombia(González-Vélez, Melo-Arévalo, and Martínez-Londoño 2019) and Argentina (Artazo, Ramia, and Menoyo 2021) in the last decade are particularly significant. Since 2020, for example, co-ordinated legal challenges by *Ni Una Menos* (Argentina) and *Causa Justa* (Colombia) campaigns, part of the *Marea Verde* (Green Wave), have led to the decriminalisation of abortion.

Abortion politics is Latin America is increasingly depicted as emblematic of how feminist movements can reverse seemingly entrenched, anti-abortion restrictions through the law(González-Vélez, Melo-Arévalo, and Martínez-Londoño 2019). It is unsurprising then that, at a time when established legal protections of abortion risk being dramatically eroded in the US, that feminist activists and commentators question what can be learnt and adopted from Latin American feminist abortion legal activism. However, to us as scholars of feminist abortion activisms in spaces and places where histories of restricted access to abortion are well established, a feminist response which pursues a renewed state provision of abortion and commitment to its protection solely through legal frameworks is problematic. It neglects the barriers to abortion entangled with but outside of the law(Amado et al. 2010; Bloomer, Pierson, and Estrada-Claudio 2018). Activisms targeted at addressing reproductive injustice and expanding abortion access, including the Latin American Green Wave, have emphasised that the law is only one technology of the un-caring state(Stifani et al. 2018). These activisms have highlighted how historic and continuing intersection of law, medicine, and policy results in states of *uncare* with regard to abortion(Ramos Jaraba et al. 2020; Svallfors and Billingsley 2019). By state of uncare we mean a context where state-led systems of care and care policies are orientated towards restricting abortion and where those who access abortion and those who facilitate it face reprisals. To address abortion uncare, these activists argue, it is important to engage with and disrupt the combination of discourses which govern reproductive health.

Using insights from research in Peru, we outline how activists have engaged with and disrupted the state of uncare for abortion. We focus on *acompañamiento* a feminist political praxis which constructs, transformation-orientated ‘infrastructures of abortion care’(Bercu et al. 2022). These political projects challenge the factors which underpin the restrictive politics of abortion in Peru through collective and holistic caring infrastructures outside the state. In doing so, it underscores the need to address the more complex problem of reproductive governance and the limitation of reproductive autonomy that is reflected in restrictive legislation. It is this project, we argue, that should guide the US feminist response to *Dobbs.*

Overall, the paper contributes to both discussions on care generally and abortion and reproductive justice specifically. For care scholarship, the paper furthers the project of relocating care theory-building from Global North/minority world histories and experiences(Raghuram 2012). The analysis of care woven through this paper has emerged from Global South/majority world praxes shaped by Global South/majority world contexts. The paper thus develops on emerging literature addressing what Raghuram (2012) provocatively refers to as feminist care theory’s “Global North problem”, or the dominance of research interventions based on the dynamics of care in the Global North. This literature does not discount care research and feminist writing on care based on and in the Global North. Rather it argues that feminist care research needs to relocate its analysis to Global South contexts.

Central to Raghuram’s contention, one which is arguably intentionally polemical, is that robust conceptualisations of care produced through analysis of Global North contexts may be ill-suited to the diverse histories, geographies, and relationalities of care beyond the Global North. Raghuram points to two components of the ‘problem’. First, how and by whom care is practiced differs between the minority and majority worlds. This is overtly recognised in decolonialist, majority world-focused writing on kinship and familial relations including the burgeoning literature on African and South Asian Aunties(Khubchandani 2021). This writing, derived from cultures outside the Global North, both displaces maternal relations, the centrepoint of early care ethics literature, and presents care practices as complex political entanglements which govern and disrupt.

Second, care in the Global South is shaped by markedly different histories than the Global North. The origins of some of these histories lie in the minority world, most obviously colonisation and the spread of colonial capitalism through imperial projects(Lutz 2018). Scholars writing in, on, and to minority world contexts have noted this relationship through, for example, recognition of global care chains(Lutz 2018). That said, there is limited consideration of the effects of development agendas applied to the majority world on care. Part of the problem, consistent with Raghuram’s argument,here is the application of a minority world understanding of policy without adopting a majority world analytic gaze. This has pronounced implications for reproductive care policy, as we will return to below, where the orientation towards expanding contraceptive access based on a minority world association of contraception with reproductive autonomy does not consider how this policy can marginalise abortion(Nandagiri 2021; Suh 2018).

For us as scholars working across care and feminism, the key project of feminist care theory should not be identifying and documenting a Global North problem but exploring how relocating to the majority world can help move feminist care forward. Through relocation, care research can, we argue, foreground the different potentialities of care the Global North can learn from the Global South. The majority world phenomena of *acompañamiento* is useful for feminist care scholars as it illustrates how care politics can draw attention to larger questions regarding the effects of macro and micro care interventions on the forms of care supported, under what circumstances, and with what contingencies(Robinson 2013). By exploring the perspectives and engagements of *acompañante* activism we can centre intersecting questions regarding how bodies are governed by care frameworks, how particular forms of care become marginalised, and how infrastructures of care can transform how we imagine care. This use of care as an entry point for larger discussions about marginalisation and governing in this paper resonates with Robinson’s (2011, 2013) and Tronto’s (2010) arguments about how we need to further our understanding of the politics of care. This includes the stratification of who cares and who can receive care along classed, raced, and gendered lines.

In terms of the contribution to abortion and reproductive justice specifically, through detailing the care politics of *acompañantes* (those who accompany people through their abortions) we contend that these Latin American care politics show that the substantive riposte to a state project of abolishing abortion care should be to generate infrastructures of abortion care beyond the state. This is a particularly timely intervention that will be of use outside of Latin America. Countries such as the Unites States and Poland become more actively uncaring regarding abortion access and by taking care scholarship beyond the Global North we can open up spaces for connection and solidarity.

The paper is organised in four sections. First, we provide a brief overview of our methodology, locating our analytic frames, and detailing data used to advance our arguments. Second, we outline the politics of abortion care in Peru. Third, we present the feminist care activisms that have emerged within and as a result of this political context, underlining how *acompañantes* have constructed an infrastructure of abortion care in Peru through the bringing together of actors, technologies, and strategies. The fourth section then explores the ethic of care than underpins and shapes this infrastructure of abortion care. We show how *acompañantes* provide abortion care that differs from minority world care models in three key ways: care as beyond the state, care as holistic, and care as collective. The article concludes by arguing that *acompañante* carework is instructive both in terms of expanding how we understand feminist care activism and in relation to the potential direction of abortion care activism in the Global North/minority world.

## 2 Methodology

We have attempted to adopt certain key aspects of feminist and decolonial approaches. Our analysis begins within the Latin American historical context, specifically that of Peru. Theoretically, this constitutes a relocation of care consistent with still nascent critiques of care theory as dominated by minority world perspectives. We have selected Peru as representative of a state that is actively uncaring with regarding to abortion.

As a general principle, this paper’s arguments, the underpinning data, and its theoretical approach reflect a commitment to feminist and decolonial methodologies. That said it is important to recognise our positionalities – two of the authors are from universities in the United Kingdom and, while we have tried to maintain dialogues with the types of political movements we discuss here, we are not members of those movements. One of the authors has tried to support feminist abortion activism in Ireland but does not claim an activist identity. One of the authors is based in a Peruvian university and has participated in feminist abortion activism, although does not currently recognise herself as an activist. We have not used data collected through participatory or co-productionist approaches. We cannot therefore claim that our methodological approach is unequivocally a decolonial feminist one.

That said, the fact that this paper is written in English for an academic publication based in the Global North is resonate with a critical component of decolonial theory. Our intention is not to tell the story of the Global South but to centre the Global South as a site of learning for a Global North audience(Vázquez 2009; Motta 2019). Through our analysis here, we aim to challenge and address progressive logics of reproductive health which dominate the Global North political landscape. First, we disrupt the positioning of majority world/Global South abortion activism which works outside the state, as the Peruvian activists we centre here do, as participating in acts of desperation. This conceptual disruption echoes the arguments relating to self-managed abortion (SMA), a practice which has itself been driven forward by Global South activists in Latin America. Abortion outside the state, within SMA literature, is a reclamation of bodily autonomy(Erdman, Jelinska, and Yanow 2018). Second, we contend that policy interventions financed and celebrated by the Global North as mechanisms for enabling greater reproductive decision-making have not facilitated reproductive autonomy. We highlight the promotion of family planning and expansion of a medicalised, clinician-led model of abortion care as particularly problematic. The former, following Nandagiri and others (Nandagiri 2021; Svallfors and Billingsley 2019), has marginalised abortion care; the latter, has displaced abortion from the community to the clinic.

In terms of data used, this paper primarily draws on empirical research in Peru, conducted between November 2020 and March 2021. This consisted of 25 in-depth, open-ended interviews with people involved in abortion access and activism, particularly *acompañante* carework. Purposive sampling was used to select a focused group of experts on the topic of abortion provision in Peru and participants were contacted through already-existing research relationships or cold-contacted by email addresses that were found online. The interviews took place on the video conferencing software ‘zoom’ due to the social distancing restrictions enforced during the covid-19 pandemic and they were conversations that covered abortion access in Peru, barriers to provision, routes to informal access and much more, depending on the interviewee.

This research project received ethical approval from [redacted for peer review] and participants were always given the option of full anonymity. All data is stored securely and accessible only by the research team. The interviews all took place in Spanish which were then transcribed and coded by the researchers. Quotes used here were translated from the original Spanish into English by the authors.

## 3 Peru and the politics of abortion

**Figure 1:**
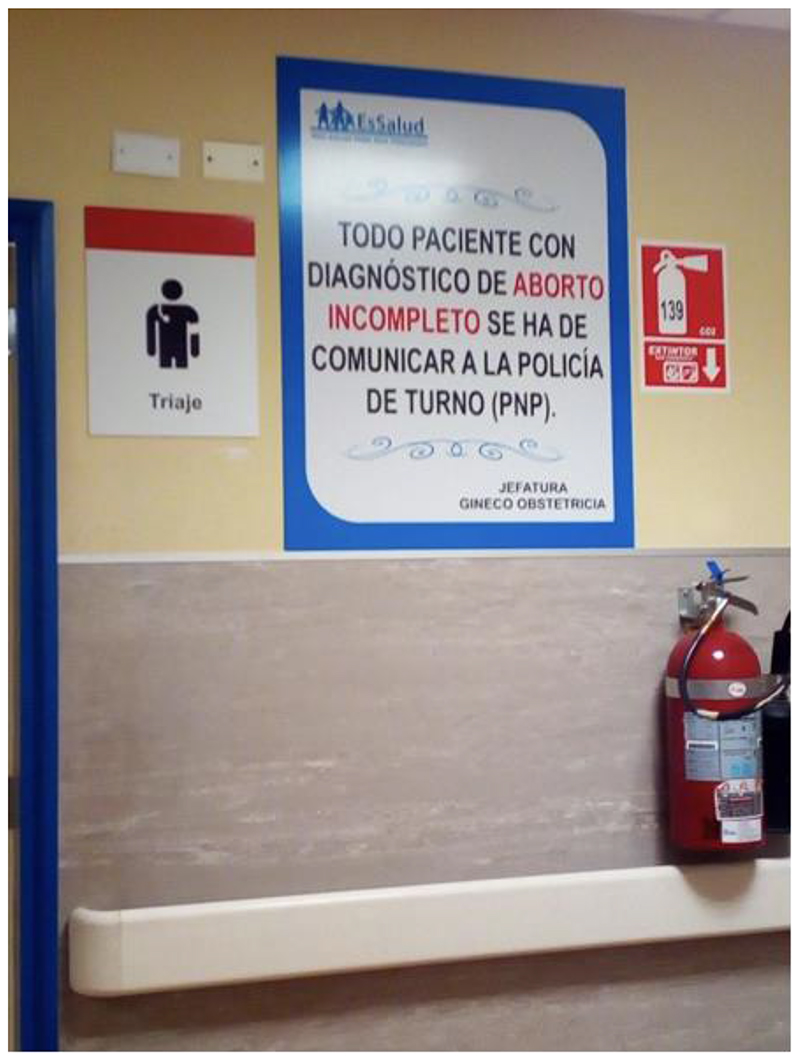
A poster a public hospital in the Abancay region of Peru warns that “every patient diagnosed with an incomplete abortion must present themselves to the police on duty”. In 2017, the poster was removed after public outrage on social media.

Clandestinity and unsafety define most people’s experience of abortion in Peru.^1^ Although first-trimester abortion is considered one of the safest medical procedures when performed in appropriate settings, clandestine conditions increase the chance of associated hospitalizations and maternal deaths.^2^ Furthermore, the health burdens of unsafe abortions unfold along sharp class and ethnic cleavages. Poor and indigenous women disproportionately suffer the potentially fatal consequences of the combination of severe abortion laws and lack of provision for abortion (Singer 2019; Wurst 2012). In addition to these health burdens, it is the women who suffer complications from unsafe abortions and are hospitalized as a result who face the greater risk of judicial punishment as the legal chain of prosecution often starts by the public hospital staff turning women to the authorities, as Figure 1 illustrates (Salazar 2019). The Peruvian state not only denies abortion as an essential form of care, but actively punishes and prosecutes women and pregnant people who attempt to interrupt their pregnancies.

Peru is thus reflective of a state of abortion un-care. Anti-abortion legislation is the most visible representation of the state of uncare and most obvious explanation for abortion’s clandestinity in Peru. Peruvian abortion law constitutes a hyperrestrictive environment (AUTHORS; (de Londras 2020)). The Peruvian criminal code criminalises abortion and a woman can be sentenced to up to two years in prison for accessing an abortion while anyone who performs an abortion can be sentenced to one to five years in prison (Cámara-Reyes, Obregón-Gavilán and Tipiani-Mallma 2018).While ‘therapeutic abortions’ have been permitted since 1924 and are currently allowed if the pregnancy poses as a high risk to the mother’s health and wellbeing, guidelines are poor and such abortions are highly difficult to access in practice, meaning Peru has one of the most restrictive abortion frameworks in Latin America (Motta and Salazar 2019) As in other jurisdictions in Latin America, those suspected of procuring abortion services illegally, including patients presenting with miscarriage and pregnancy loss, have been pursued through the courts. As the image above illustrates, practitioners in clinics have been co-opted by the legal project restricting abortion. Such legal frameworks and examples of anti-abortion lawfare (Enright, McNeilly, and de Londras 2020) are visible across Latin America and the Caribbean. In Uruguay, Honduras, and El Salvador, for example, patients presenting with miscarriage have face criminal charges and even imprisoned under anti-abortion legislation.

However, the anti-abortion legal framework is only one way that the Peruvian state has constrained abortion access. To understand fully the state of abortion un-care, it is essential to take the broader context of reproductive politics into account. Peru represents a country where successive, persistent political discourses and interventions have resulted in a situation where abortion is inaccessible even when it is legally permissible(Rousseau 2007). The clandestinity of abortion in Peru is as much the result of this inaccessibility as the legal prohibitions, a phenomenon discussed by Morgan and Roberts (2012) through their model of reproductive governance. In Peru, the individual experience of abortion un-care is rooted in co-existent state projects, national and transnational, relating to family planning, maternal health, and the medicalisation of abortion.

The use of family planning initiatives to minimise abortion has particular relevance to Peru where a combination of Catholic Church-led natalist concerns about “the improvement of families as Catholic communities”(López 2008:58) and reliance on international donors such as USAID(Ewig 2006) worked to explicitly orientate reproductive health provision towards contraception and reducing ‘unsafe’ abortion rates(Rousseau 2007). Doctrines of fertility control are embedded within reproductive health policy and investment in Peru, and discourses from the 1970s onwards have actively connected ‘good motherhood’ with fertility control, the continuation of pregnancy, and the provision of material support for children (Rousseau, 2007; López, 2008). The target populations of these programmes and discussions were predominantly already marginalised communities – rural, poor, and Andean specifically – and there is extensive evidence of the escalation of reversible family planning to coerced and non-consensual mass sterilisation of these groups by state-backed agencies (Boesten, 2014). This escalation is reflected most overtly by the state-run sterilisation campaign under the Fujimori administration in the mid-1990s. The combined result of these state projects is the reduction of investment for abortion care, the stigmatisation of those who seek abortion as failed reproductive citizens, and the formation of a political ideology that favours reproductive control above reproductive autonomy.

The genealogy of abortion un-care and clandestinity in Peru resonates with global histories of reproductive justice. In the US and globally, the emerging restrictions on abortion have been preceded by conservative emphases on contraception and good mothering. National and global reproductive health projects have prioritised pregnancy prevention and maternal health support (Nandagiri, 2022). These have facilitated the marginalisation of abortion within the spectrum of reproductive care provision, a phenomenon referred to by Suh (2019) and Amleling (2015) as reproductive stratification. As Suh and Nandagiri note, despite substantial investment in reproductive health by transnational organisations, and statements on the importance of reproductive health’s importance such as the 1992 Cairo Declaration, clandestinity and un-care remain a common experience for abortion seekers. The provision and availability of abortion services nationally and locally is often unequal, creating insurmountable cost burdens and resulting in abortion travel(Bloomer, Pierson, and Estrada-Claudio 2018). These inequalities intersect with other social determinants of poor health access and compound uneven political economies and geographies of abortion. Encounters with stigmatising attitudes from health professionals are frequently reported in research (Suh, 2018). These attitudes are worsened by reproductive health interventions focused on fertility control as abortion seekers are cast as irresponsible(Morgan and Roberts 2012).

What is distinct about Peru, and Latin America more broadly, is the response of feminist activism. Although Latin American feminist activists have responded to abortion un-care through, like minority world activists, demanding the state guarantee abortion rights and highlighting the uneven political economies of reproductive (in)justice, a prominent strand of feminist activism – *acompañante* activism - has emerged that foregrounds providing abortion care in a way that challenges the discourses underpinning abortion un-care. This involves *acompañantes* engaging in practical support activities that are visible in minority world movements, including providing information, supporting abortion travel, and advocating for an appreciation of non-legal barriers to abortion. Yet, the totality of *acompañante* activism is, as Braine (2022) notes, incomparable to feminist abortion care activisms in the minority world. The fundamental difference is that *acompañantes* not only demand the minimisation of legal barriers to abortion care but also engage in the formation of new infrastructures of abortion care. These infrastructures disrupt minority world concepts of safe abortion care as medicalised and provided via state institutions, producing a collective and holistic infrastructure of care beyond the state(Bercu et al. 2022).

The emergence of this activist pursuit is intertwined with the genealogy of abortion un-care outlined above. The family planning ‘revolutions’ and the reliance on conservative international investment indicate that the Peruvian state cannot be fully trusted to protect reproductive autonomy. Moreover, the history of abortion law in Peru, as in other Latin American countries such as Venezuela and Colombia, indicates that liberalisation of reproductive health has frequently been underpinned by anti-abortion projects(Ewig 2006). Peruvian law in relation to contraception was increasingly subject to liberal reforms throughout the 1970s and 1980s; by comparison Peru’s draconian abortion laws have remained largely untouched(Rousseau 2007). Investment in maternal health and contraception, including coercive contraception, dominate the policy landscape. The state emphasises the need to address abortion as a public health and safety problem, an approach which Suh (2018) argues reinforces the reproductive stratification outlined above.

The Peruvian activist response is also connected to a problematisation of the medicalization of abortion as exemplary of colonialist projects intent on establishing and reinforcing medical hegemony. Toward the end of the nineteenth century abortion became ‘medicalized’ in minority world contexts in that it was only to be performed by medical professionals and with this came the implementation of laws prohibiting traditional and community-based abortion care (Bloomer et al, 2019). These medicalized abortions prioritize state-sanctioned provision, medical procedures performed by specific medical professionals, and an individualized form of care between the abortion-seeker and the medical professional. Medicalised abortion care models emphasise individual safety, associating care outside of clinical care frameworks as potentially riskier and less safe. The abortion experience itself is reduced to an individual, reproductive event that should be managed by clinicians (Sheldon, 1997).

Decolonial theorists such as Vazquez (2009), argue that the ascendancy of medical models reflects a hegemonic project of obliviating traditional forms of care and knowledge in the majority world. Global investment for safe abortion initiatives, include post-abortion care, reinforce the hegemonic position of medical, clinic-based professionals (Suh, 2018; Erdman et al, 2018). Even policies, such as the Peruvian ‘task-sharing’ programme with Andean community birthing care, which present themselves as interested in expanding care have been critiqued for this reason. Analysis of the implementation of the Andean task-sharing initiative indicate that, during implementation, care is decentred in favour of medicalisation(Guerra-Reyes 2016; 2009). Those who opt to access care outside clinics are stigmatised and discriminated against by health professionals. Literature on bodily and reproductive autonomy considers such experiences as manifestations of obstetric violence as decision-making becomes denied. This results a sense among indigenous communities and activists working towards individual reproductive control that the state’s intention is to erase their reproductive autonomy.

Having contextualised the situation of clandestinity and state harm in Peru we now explore how a diverse range of feminist groups and collectives have constructed what we term an ‘infrastructure of abortion care’ to provide caring abortions against the backdrop of this history of reproductive control and state un-care.

## 4 Building feminist infrastructures of abortion care in Peru

Peru’s hyperrestrictive environment means that most abortions are performed ‘clandestinely’, beyond the eyes of the state. Clandestine abortions include abortions supported by health providers working in clandestinity outside clinics or formal medical settings (e.g. hospitals). However, this does not mean that all abortions accessed in Peru, or Latin America generally, are unsafe or uncaring. As Erdman et al (2018) note, while clandestine abortion is frequently positioned as dangerous, empirical health research challenges reading clandestinity as synonymous with unsafety. The problem with clandestinity is that it is challenging to know where and how to access an abortion in a safe, cared-for way if one cannot use ‘formalised’ routes (i.e. in hospitals or medical clinics). In response to this challenge, a series of actors, including health workers, mobilize to facilitate access to caring abortions. In the remaining part of this section, we show how individuals, groups, and organisations, alongside technologies and strategies, are woven together to forge - and continually recreate - infrastructures of abortion care. First, we explain the terms ‘infrastructure’ and ‘infrastructure of care’.

‘Infrastructure’ has become a fashionable term in recent years. While it has been defined in many different ways both within and outside academia and has defied any fixed definition (Fourie 2006; Wilson 2016), there is a growing body of scholarship within what has been termed the ‘infrastructuralturn’ (Amin 2014). Early work on infrastructures considered them to be “by definition invisible, part of the background for other types of work” (Star 1999, 377) and “frequently mundane to the point of boredom”, but Star’s important contribution here was to call attention to how overlooked infrastructures, whether sewers, power supplies, or bureaucratic forms, help us interrogate questions of power and justice (ibid). This work also focused on how infrastructure is not a discrete thing and of itself but is always a *relation* (Star and Ruhleder 1994). Such scholarship led to an understanding that infrastructures are not the background of life upon which things run, rather they are political and we need to understand how infrastructures are brought into being and continually re-made (Bowker and Star 2000). As Lauren Berlant has argued (2016, 393), “infrastructure is defined by the movement or patterning of social form. It is the living mediation of what organizes life: the lifeworld of structure”. It is dynamism and the potential for restructuring then, that is the common thread that runs through current conceptualisations of infrastructure.

A very recent development in scholarship on infrastructure has been the coining of the term ‘infrastructure of care’ (Power and Mee 2020; Alam and Houston 2020; Odendaal 2021). Drawing on the work of Star (1999), Power and Mee (2020, 485) “conceptualize infrastructures not as pre-figured objects or necessarily public, capital goods, but as dynamic patterns that are the foundation of social organization” in their work on housing as an infrastructure of care. In her work on South Africa’s covid-19 response, Odendaal (2021, 391-392) uses ‘infrastructures of care’ to refer to “the data, technology and human agency that contribute to the care landscape emerging from the virus response”. Alam and Houston (2020), meanwhile, consider care as ‘alternate’ infrastructure to focus on the ordinary and the intimate, and the role of everyday, non-institutional care spaces. This nascent work is providing a framework for understanding the organisation of care that includes people but also takes into account materialities, technologies, systems, and governance. These all shape what kind of care is possible, if at all. Importantly, Power and Mee (2020) highlight how values become coded into infrastructures which then (re)produces social difference.

If current conceptions of ‘infrastructures of care’ provide a framework for understanding the organisation of care that includes people but also takes into account materialities and technologies, we define ‘infrastructures of abortion care’ as a set of relations between actors, technologies, and strategies that are brought into being by an interest in the embodied and emotional wellbeing of the people seeking to have an abortion. These infrastructures of care create possibilities for abortion in restricted contexts by expanding the paths of action available to people, providing a level of regularity and predictability of the abortion experience, and serving as a source of practical and emotional support throughout the abortion trajectory. This infrastructural work, following Alam and Houston (2020), also reimagines the possible shape of abortion care. The next section will outline how the infrastructures generated by *acompañamiento* at the margins of an uncaring state, reimagine abortion care. As a preface to this, here we explain the actors, technologies, and strategies that generate this infrastructure of abortion care.

The actors that constitute the infrastructures of abortion care are not uniform and are mainly differentiated by the actors’ level of organization, the technologies and knowledge they deploy, and the strategies they use to interact and respond to clandestinity. Although the limits between them are blurry and overlapping, we can broadly identify three type of actors that build infrastructures of care in Latin America: family and friends, healthcare practitioners, and *acompañantes.* First, family and/or friends that accompany people seeking to terminate their pregnancies are often the only source of help and support in Latin America (Erviti 2005; AUTHORS). While social networks can reduce associated physical, mental or emotional burdens, and facilitate access to timely medical care in cases of an emergency, their help can also be experienced with ambiguity or, at worst, can represent an additional source of stress, for instance in the cases where they oppose the pregnant person’s decision. Second, there is an extensive network of healthcare practitioners that provide safe surgical abortions in Latin America. Throughout the 1990s, a growing network of healthcare providers were trained to effectively deliver abortions using manual vacuum aspiration (MVA). These health providers included not only physicians but also registered midwives, nurses, nurse technicians and even traditional midwives -a cadre of health professionals that provide the bulk of reproductive healthcare in Latin American as in other developing countries, especially in small cities and rural areas (Berer 2009). Once trained, these same professionals could themselves implement projects to train others in even more remote areas, expanding the network much further. This has had a big impact on the enlargement and decentralization of care sites, increasing access to safe abortion services.^3^

Third, and the actor that is the primary focus of this paper, there is a growing number of feminist networks of activists and *acompañantes* that facilitate access to self-managed abortions in Peru. The first *acompañante* network emerged in Lima, Peru’s capital, in 2009, and after the massive mobilizations for *Ni Una Menos* (Not One Woman Less), against the multiple forms of gender-based violence, that upsurged in 2016, *acompañante* networks have spread within and outside Lima. *Acompañantes* are groups or individuals who provide accurate information and support in self-managing an abortion alongside emotional support, legal guidance, practical resources, and post-abortion care (Veldhuis, Sánchez-Ramírez and Darney 2021). A*compañantes* are typically more involved in the process than other groups who just provide information about abortion, sometimes offering in-person accompaniment.^4^ In recent years, in Latin America, *acompañantes* have become pivotal actors in establishing infrastructures of abortion care. Furthermore, as we outline later in this paper, in creating infrastructures of care *acompañantes* foreground a transformative imagining of abortion care as a holistic and collective experience which can exist beyond institutionalised, state-governed spaces.

Self-managed abortions, as supported by *acompañantes,* consist of taking abortion pills, commonly referred to as a ‘medical abortion’. The dose may consist of a combination of mifepristone and misoprostol or just misoprostol. Misoprostol is a medication that was designed to treat stomach ulcers but women in Brazil realised its abortifacient properties and developed a safe and effective protocol to use it to end pregnancies. It is most effective when used in combination with the medication mifepristone but this is harder to access in Latin America because it is only used for abortions. With the correct information on how to use the pills, medical abortions are safe and effective and may be preferred by some abortion seekers as they are more affordable and less invasive (Moseson et al. 2020). They also afford greater reproductive autonomy and allow abortion seekers to avoid domains where they may have to defend their decision making or which they have historically experienced as sites of reproductive injustice and control (Erdman et al, 2018). Yet the existence of this pill does not guarantee easy access and, on top of that, without accurate information on how to use them, they could be not only ineffective but in extreme cases, risky. As one interviewee explained, “so it [misoprostol] seems great to me when it's used properly, with good information, with good medical support for women, but when all that is not there then it is also putting them at risk”. The role of *acompañante* groups is therefore to build an infrastructure that provides access to the pills themselves as well as access to information on how to use them safely. These are the technologies and strategies in our definition of an ‘infrastructure of abortion care’.

The technologies of *acompañante* abortion care are elements that are utilized in order to make abortion care possible. In terms of providing access to abortion medications this might be the postal system or public transport. In order to provide information about using the pills a range of technologies are used such as hotlines where callers can access accurate and up-to-date guidance on how to safely seek an abortion or handbooks which explain how to self-manage an abortion using text and visual guidance (Drovetta 2015). Social media and instant messaging services have also become crucial technologies and our interviewees reported using encrypted, secure platforms such as Signal and Telegram to avoid surveillance. These allow *acompañantes* to remain anonymous and offer a range of communication including text-based messaging, voice calls, video calls, and the sharing of images. This latter medium has been particularly important during the covid-19 pandemic when *presencial* (in-person) accompaniment became challenging or impossible and *acompañantes* shifted their carework to be virtual. These technologies enabled the sharing of photos and videos so that *acompañantes* could share their expertise on whether the amount of blood looked excessive or whether the gestational sac had passed. Such technologies form one strand of the *acompañante* infrastructure of abortion care.

These technologies are enabled by strategies that facilitate the movement of abortion pills and information of how to use them. For example, one group explained how they created ‘heat maps’ that noted different pharmacies and provided information on which sold misoprostol, how much they charged, and whether they required a prescription. Another strategy is to use codewords for misoprostol and many groups have their own preferred terminology, from sweets to cupcakes, communion wafers to ingredients. A third strategy is the building of strategic relationships. Some groups will work closely with or be part of broader coalitions involving medical professionals, legal advocates, health researchers, and community leaders. This means they can access support but also material resources as some international organisations are able to donate mifepristone and misoprostol directly to activists in Peru and Latin America more broadly. A final strategy is to provide information about what to do if an abortion seeker is questioned by the authorities, perhaps if they experience complications and need medical attention. If misoprostol is administered buccally it is impossible for a medical professional to know that an abortion was intentionally provoked and so *acompañantes* provide training and ‘scripts’ on what to say so that it appears to have been an unprovoked miscarriage. In all, a range of strategies are employed to provide safe and effective abortion care that protects those having the abortion as well as those who support them.

In this context, a diverse range of feminist groups and collectives, formal and informal, have constructed an infrastructure of abortion care to minimize these risks and to provide caring abortions. These actors work to mobilize the pills, disseminate information about how to safely administer them, and provide care for those undergoing an abortion process. Through these strategies, relationships, and processes, an infrastructure of pills, information, and support are pulled together by these actors to provide abortion care in an uncaring state. We next turn to the ethic of care that drives and shapes this infrastructure of abortion care.

## 5 Developing abortion care through *acompañante* carework

Within an un-caring context, *acompañantes* prioritise abortion care as their central activist project. Feminist infrastructures of care offer radically different forms of abortion provision through rejecting assumptions that safe abortion can only be provided by the state, through clinical frameworks(Erdman, Jelinska, and Yanow 2018). *Acompañantes* generate infrastructures of safe abortion care that are beyond the state, holistic rather than just medical, and collective. Here, we use these three elements to first outline what distinguishes *acompañante* carework as a political project and second to suggest what care theory and feminist activists can draw from the *acompañantes’* politics.

First, *acompañante* carework shows that it is possible to provide safe and effective abortions beyond the state. While there are feminist organisations and activists who argue for the state to step-in and provide abortion care, the urgent need for safe abortions in the present means that *acompañantes* recognise the need to provide abortion care outside of state frameworks. Indeed, our interviews with *acompañantes* in Peru showed some frustration over what one called the ‘liberal approach’ to fighting to change the abortion law. As an interviewee emphatically stated, self-managed medical abortion is necessary now because “women continue to die … I’m not going to wait... for congress [to legalize abortion].” Another explained :

Sé que aquí no va a ser legal no sé en cuántos años, pero creo que si acompañamos a una o a dos estamos ayudando un granito en que no pase algo terrible, ¿no?

I know it's not going to be legal here, I don't know in how many years, but I think that if we accompany one or two we're helping a bit so that something terrible doesn't happen, right?

However, these actions have bred recognition among activists that the Peruvian state has been a harmful actor in relation to reproductive autonomy(Rousseau 2007). As a response, *acompañantes* have actively created alternative pathways for feminist, empathetic abortion care. One interviewee described her journey to creating abortion access in the present and how this led her to engage with feelings towards abortion care. She explained that she had previously thought that the best strategy was to fight for ‘legal and safe’ abortion in Peru until a friend of hers asked her what that meant in the meantime. From there she began the process of confronting her emotions of shame and fear around accompanying abortions. As these examples show, claims that care should be the responsibility of institutions and the state do not work in the example of abortion care in Peru when abortions are urgently needed in the present and when the state has so often been harmful. *Acompañante* groups create alternative infrastructures of abortion care beyond the state to address an immediate need and to disrupt the view that abortion should only be provided by the state.

Second, *acompañante* groups are further distinguished by their rejection of clinic-based, medicalized abortion care as the *only* safe form of abortion care. These groups do not wholly reject medical practitioners; many have medical professionals inside the group who are able to offer knowledge and support. Nevertheless, *acompañantes* come from a more expansive place of care, underscoring the holistic, relational qualities of positive abortion care that encompass but go beyond the medical interruption of pregnancy. One *acompañante* described her abortion carework as both practical and emotional, which together form the *‘nivel holístico’* [the holistic level]. Another called it *‘acompañamiento integral’* [comprehensive accompaniment] because they would sit down and talk and ask “what do you feel? What do you want?”. This is tailored care that takes into account the full lives of those seeking accompaniment in what one *acompañante* group call *‘acompañamiento diferenciado’* [differentiated accompaniment], “that is to understand that we do not all live in the same way, nor do we see the world in the same way”. This changes what a ‘good’ or ‘safe’ abortion consists of. For example, one interviewee defined ‘safe abortions’ as”

No solo en el sentido médico, del cuerpo, sino seguros en el aspecto de la salud mental y de las emociones.

Not only in the medical sense, of the body, but safe in the aspect of mental health and emotions.

In order to provide this type of holistic care, *acompañante* groups build their infrastructure of care differently. This might mean in terms of who they have within their collective as groups often have psychologists or therapists within their team to provide support. It also dictates their protocol and what they offer abortion seekers. As one interviewee explained:

Sí, el saber más técnico, pero también el acompañamiento humano, acompañar el duelo, la toma de decisiones, lo que implica esto, hacer que la mujer no esté sola. Entonces, básicamente información, acompañamiento en todas sus dimensiones, ¿no?

Yes, the more technical knowledge, but also the human aspect of accompaniment: accompanying the grief, the decision-making, what it all means, making sure that the woman is not alone. So, basically information, accompaniment in all its dimensions, right?

Therefore, *acompañante* carework is formulated differently from the majority of medicalized abortion providers. Medical knowledge and expertise are important but emotional support is embedded in the process from the beginning and the whole health, mental and physical, of the abortion seeker is considered at all stages to create a holistic model of care that can extend beyond the procedure itself.

Third, *acompañante* groups construct an infrastructure of abortion care where abortion is embedded within communal and community relationships. Again, this stems from a recognition of the negative impacts of medicalising abortion. Medicalising abortion can reinforce the social stigmatisation and marginalisation of abortion by moving abortion out of community and collective relationships(Suh 2019). It can also overlook the problematic attitudes of providers in clinics towards abortion and reproductive health (Halfmann, 2012) and decentre the importance of creating atmospheres of trust and dialogue between carers and the cared-for (Tronto and Fisher, 1990). While the expansion of self-managed abortion, driven by Global South activists in Brazil (Baum et al. 2020; Diniz, Ambrogi, and Carino 2021; Bloomer, Pierson, and Estrada-Claudio 2018), have led to the expansion of telemedicine and abortion ‘at home’ in the minority world, an expansion that escalated rapidly during the Covid-19 pandemic(Lohr 2022), medical models have not been displaced. ‘At home’ abortion care, ‘community-models’, and telemedicine in countries such as the UK, US, and Australia remains clinician-led, underscored by concerns of risk and safety. These forms of community care are still medicalised.

By contrast, ideas around mutuality, trust, and the collective underpin *acompañante’s* approaches to community-based care(Braine 2022). The collective is an important way to challenge the traumatic, harmful violence of clandestine abortion in Peru. This includes feelings of isolation and shame. For example, an *acompañante* recalled one of her friends who had an ‘abortion shower’, surrounded and supported by friends and this, despite the physical pain of the process, “meant that it was not a traumatic experience”. Engaging the collective in abortion care can have transformative effects on how *acompañantes* understand the problem of clandestine abortion as more than an issue of safety. Hearing about this friend’s experience was what convinced our interviewee that it is possible to have an abortion free of guilt and feelings of sin. Another interviewee explained, “the worst thing [about having an abortion in Peru] is the clandestinity and the fear of not knowing who to turn to”. Furthermore, a different interviewee, reflecting on her own abortion in Peru and others that she had accompanied that:

como colectividad entendamos que el aborto no es un proceso traumático en sí mismo sino que todo el contexto lo hace traumático

as a collective we understand that abortion is not a traumatic process in itself but that the whole context makes it traumatic

To create this non-traumatic, collective space often entails *acompañantes* bringing their own personal experiences into the caring process. One *acompañante* explained that in her group all the accompaniers had had an abortion themselves so that they can explain to those they accompany that they know how they feel. Another explained:

dependiendo también de la mujer y cómo se muestre ella, también salen las experiencias personales como ‘mira, por ejemplo, en mi caso fue así, y todo salió bien, que no sé qué’; esto nos acerca, nos acerca un montón. Lo cual no quita que todas las experiencias sean gratas.

also depending on the woman and how she shows herself, personal experiences also come out like 'look, for example, in my case it was like that, and everything went well, I don't know what'; This brings us closer, brings us a lot closer. Which does not mean that all experiences are pleasant.

Moreover, this collective nature of care is facilitated through group sharing sessions where people who are seeking an abortion or who have had one can vocalize their experience and hear the experiences of others. This is with the clear purpose to combat stigma, raise consciousness, and make abortion care a collective rather than an isolated experience.

*Acompañante* carework in Peru, as in other Latin American jurisdictions, is thus more than providing a route to an effective abortion through challenging either legal barriers to abortion or expanding state provision in clinical settings(Bercu et al. 2022; Baum et al. 2020). It necessitates creating a supportive environment in which that abortion takes place. While minority world discourses have predominantly focused on asking the state to assume responsibility for abortion provision, situating our analysis from Peru shows that this conversation needs to appreciate the fact that, even when care is assumed by the state, care is not always facilitated in beneficial ways. Access to abortion can been stratified along racialised, classed, and moral lines and already-marginalised communities are particularly vulnerable to this. *Acompañante* groups’ focus on abortion care as holistic challenges medicalized frameworks of abortion care that posit an abortion procedure as isolated and detached from the rest of the persons health and life. An important exception to note here is the emotional and social carework performed by abortion doulas through the abortion process in global North contexts such as North America and Northern Ireland (Chor et al. 2016; Campbell et al. 2021). Lastly, by making abortion carework a collective endeavour, *acompañantes* challenge assumptions that abortions that take place in clandestinity are necessarily traumatic and isolating. Through sharing their experiences and learning from the experiences of others, those who have abortions can be well supported and find the process personally and politically transformative. This model refutes care as a transactional, one-way process which is reflected in the term *acompañar* [accompany] which is used instead of ‘help’ by these activists to highlight that the relationship is one of learning and reciprocity (Vivaldi and Stutzin 2021). This alternative ethic of care provides important lessons for minority world contexts of uncare.

## 6 Conclusions

We began this paper by asking what the Global North could learn from the Global South. On one level, Latin America presents a set of strategic tools. These include forms of feminist lawfare that work through rights-based legal tools and technologies of providing abortion in a state of uncare such as abortion pills. At another level, one more interesting to us, Global South activisms present an opportunity for Global North pro-choice feminist to unlearn assumptions about what their request should be. *Acompañante’s* infrastructure of abortion care presents a different set of objectives for feminist activists in the US facing the reversal of *Roe.* The orientation of these Latin American activists is distinct from the orientation of pro-choice abortion activism that has been developed in minority world contexts since the mid-20^th^ century, particularly in North America. Pro-choice activism in the US has emphasised legally protecting abortion, increasing contraceptive use, minimising ‘unsafe’ abortion, and expanding provision by medical professionals through clinical frameworks. Within these minority world models of good abortion care barriers to abortion, unsafe abortion, and clandestine abortion are addressed through a combination of liberalisation; investment in health services including telemedicine, telehealth, and post-abortion care; and ensuring that voluntary contraception including long-acting reversible contraceptives (LARCs) are widely available.

Majority world countries such as Peru, have a distinctly different history with regard to abortion. As a result of legal frameworks which have been relatively unchallenged since the 1980s, abortion is almost always a crime in Peru. This means that women and pregnant people wanting to end their pregnancies must find alternative strategies and conceal their abortion from a state that actively prosecutes abortion seekers and providers. Legal pro-choice advocacy is slow and does not address these immediate concerns. Furthermore, like in the US, the barriers to abortion extend beyond the law with uneven effects. Indigenous and poorer communities experience the greatest difficulties. These barriers have been worsened by other reproductive health policies. The emphasis on family planning investment and medicalisation of abortion has marginalised abortion within the spectrum of reproductive health and positioned the decision to have an abortion outside a clinic as an act of desperation and unsafe. Communities who either have accessed or prefer to access abortion outside state institutions are faced with the problem of navigating clandestine abortion trajectories in isolation. This isolation reinforces abortion stigma and creates safety concerns.

In the face of a state that refuses to respect either the normalcy of abortion care or respect the decision to access abortion outside of clinical spaces, groups and individuals have developed forms of caring that provide safe, effective, respectful, and empathetic abortions. These activists - *acompañantes* - do not just address the limitations on state-provided or state-sanctioned abortions, but put forward a new vision of abortion care, one where the person having the abortion feels empowered and has autonomy. To appreciate the complexity of this activism, we have proposed *acompañantes* as generating ‘an infrastructure of abortion care’. The term ‘infrastructure’ allows us to think beyond just the people involved in carework to include the materialities and technologies that facilitate that care. Recent scholarship on ‘infrastructures of care’ (Power and Mee 2020; Alam and Houston 2020; Odendaal 2021) has conceptualised such infrastructures as dynamic patterns that are underpinned by values. Effective abortion care beyond the Peruvian state, in clandestinity, is constituted by technologies and strategies as well as the people facilitating the *relationships* between these. And it is a vision of empathetic, autonomous abortion futures, not just legal abortion provided through clinical frameworks, that drives this infrastructure of abortion care. *Acompañante* abortion carework is beyond the state, holistic, and collective. As shown through this paper, *acompañante* carework is, for many carers, a process filled with love. A post-Roe future, through learning from *acompañantes,* could potentially bear witness to a similar process.
